# GSK‐3β activation mediates apolipoprotein E4‐associated cognitive impairment in type 2 diabetes mellitus: A multicenter, cross‐sectional study

**DOI:** 10.1111/1753-0407.13470

**Published:** 2023-09-12

**Authors:** Yang Gao, Haitao Yu, Yanchao Liu, Zhipeng Xu, Benrong He, Honghai Liu, Yuying Wang, Yao Zhang, Yi Liang, Ying Yang, Jie Zheng, Jian‐Zhi Wang

**Affiliations:** ^1^ Department of Pathophysiology, School of Basic Medicine, Ministry of Education Key Laboratory for Neurological Disorders, Hubei Key Laboratory for Neurological Disorders Tongji Medical College, Huazhong University of Science and Technology Wuhan China; ^2^ Department of Radiology Wuhan Brain Hospital Wuhan China; ^3^ Department of Fundamental Medicine, Wuxi School of Medicine Jiangnan University Wuxi China; ^4^ Department of Neurosurgery, Tongji Hospital, Tongji Medical College Huazhong University of Science and Technology Wuhan China; ^5^ Department of Neurology Zhongnan Hospital of Wuhan University Wuhan China; ^6^ School of Medicine and Health Management, Tongji Medical College Huazhong University of Science and Technology Wuhan China; ^7^ Li‐Yuan Hospital, Tongji Medical College Huazhong University of Science and Technology Wuhan China; ^8^ Neuroscience Research Institute and Department of Neurobiology, School of Basic Medical Sciences Peking University; Key Laboratory for Neuroscience, Ministry of Education/National Health Commission, Peking University Beijing China; ^9^ Co‐innovation Center of Neuroregeneration Nantong University Nantong China

**Keywords:** *ApoE*, gene polymorphism, glycogen synthase kinase‐3β, mediation analyses, mild cognitive impairment, type 2 diabetes mellitus

## Abstract

**Aim:**

Both the activation of glycogen synthase kinase‐3β (GSK‐3β) and the presence of *ApoE ε4* genotype have been found to respectively correlate with cognitive decline in patients with type 2 diabetes mellitus (T2DM), who further show a high incidence of developing Alzheimer's disease. However, the relationship between *ApoE ε4* and GSK‐3β in the cognitive impairment of T2DM patients remains unclear.

**Methods:**

ApoE genotypes and platelet GSK‐3β level were measured in 1139 T2DM patients recruited from five medical centers in Wuhan, China. Cognitive functions were assessed by Mini‐Mental State Examination (MMSE). The association and the relationships among apolipoprotein E (ApoE) genotypes, GSK‐3β activity and cognitive function were analyzed by regression and mediating effect analyses, respectively.

**Results:**

T2DM patients with *ApoE ε4* but not *ApoE ε2* haplotype showed poorer cognitive function and elevated platelet GSK‐3β activity, when using *ApoE ε3* as reference. The elevation of GSK‐3β activity was positively correlated the diabetes duration, as well as plasma glycated hemoglobin (HbA1c) and glucose levels. Moreover, correlation and regression analysis also revealed significant pairwise correlations among GSK‐3β activity, *ApoE* gene polymorphism and cognitive function. Lastly, using Baron and Kenny modeling, we unveiled a mediative role of GSK‐3β activity between *ApoE ε4* and cognitive impairment.

**Conclusion:**

We reported here that the upregulation of GSK‐3β activity mediates the exacerbation of cognitive impairment by *ApoE ε4*‐enhanced cognitive impairment in T2DM patients, suggesting GSK‐3β inhibitors as promising drugs for preserving cognitive function in T2DM patients, especially to those with *ApoE ε4* genotype.

## Graphical abstract

Highlights

- The association of *ApoE* polymorphism with cognitive impairment in type 2 diabetes mellitus (T2DM) was inconclusive. Moreover, it is not reported whether and how glycogen synthase kinase‐3β (GSK‐3β), a crucial kinase in insulin resistance and Alzheimer disease‐like pathologies, play a role in linking *ApoE ε4* and cognitive impairment.
- We revealed that *ApoE ε4*, but not *ApoE ε2*, was a risk factor of cognitive impairment in Chinese Han population. GSK‐3β activation acts as a mediator between *ApoE ε4* and cognitive impairment.
- These data reveal a novel role *ApoE ε4* and GSK‐3β in T2DM‐associated cognitive deficits. Targeting GSK‐3β, such as inhibitor, could be a constructive strategy in preserving the cognitive function in T2DM patients but need further proof.

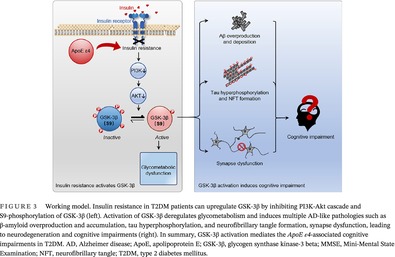

## 1 INTRODUCTION

Type 2 diabetes mellitus (T2DM) is an independent risk factor of Alzheimer's disease (AD), the most common cause of dementia in the elderly.^1^ T2DM and AD share many pathophysiological features, such as insulin resistance, deregulated glucose metabolism, peripheral oxidative and inflammatory stress, amyloid aggregation, and neurodegeneration.^2^, ^3^ The precise mechanisms by which T2DM increases the risk for AD are not fully understood, but substantial evidence links AD risk to insulin resistance and impaired insulin signaling in T2DM.^4^

Apolipoprotein E (*ApoE*) genotype is the most consistently observed genetic contributor to late‐onset AD. There are three alleles of *ApoE* gene, namely ε2, ε3, and ε4, which encode ApoE2, ApoE3, and ApoE4 protein, respectively. Large‐scale genome‐wide association and meta‐analyses suggest that the ε3 is a wildtype and the most common allele, although the *ε4* is a risk factor and *ε2* is a protective factor for sporadic AD.^5^ It is also reported that both ε2 and ε4‐carried T2DM patients seem to perform worse in cognition test.^4^ *ApoE* genotypes also show ethnic variations.^6^, ^7^, ^8^ The association of *ApoE* polymorphisms with cognitive function in Chinese Han T2DM patients and its downstream mediator have not been reported.

Glycogen synthase kinase‐3β (GSK‐3β) is a serine–threonine kinase playing vital roles in multiple processes including insulin signaling, glucose metabolism, and aging, etc.^9^ Activation of GSK‐3β functions against insulin by disinhibiting glycogen synthesis and facilitating glucose absorption.^9^, ^10^ Simultaneously, it can also induce AD‐like tau hyperphosphorylation and exaggerate neurodegeneration during accelerated aging.^11^ Elevated GSK‐3β has been found in the periphery platelet of T2DM patients with mild cognitive impairment (MCI) compared with those without MCI.^12^ Given that the GSK‐3β activity can be regulated by ApoE in vitro,^13^, ^14^ we hypothesize that the activation of GSK‐3β may play a mediative role between *ApoE ε4* and cognitive impairment in T2DM patients.

In the present study, we aimed to explore the association of *ApoE* gene polymorphism with the cognitive functions, and the downstream mediator of specific *ApoE allele(s)* in leading to the cognitive impairment in Chinese Han T2DM patients. The results reveal a positive association of *ApoE ε4* with cognitive decline in T2DM patients, and a mediative role of GSK‐3β activation between the *ApoE ε4* and cognitive impairment.

## 2 MATERIALS AND METHODS

### 2.1 Participants

**FIGURE 1:**
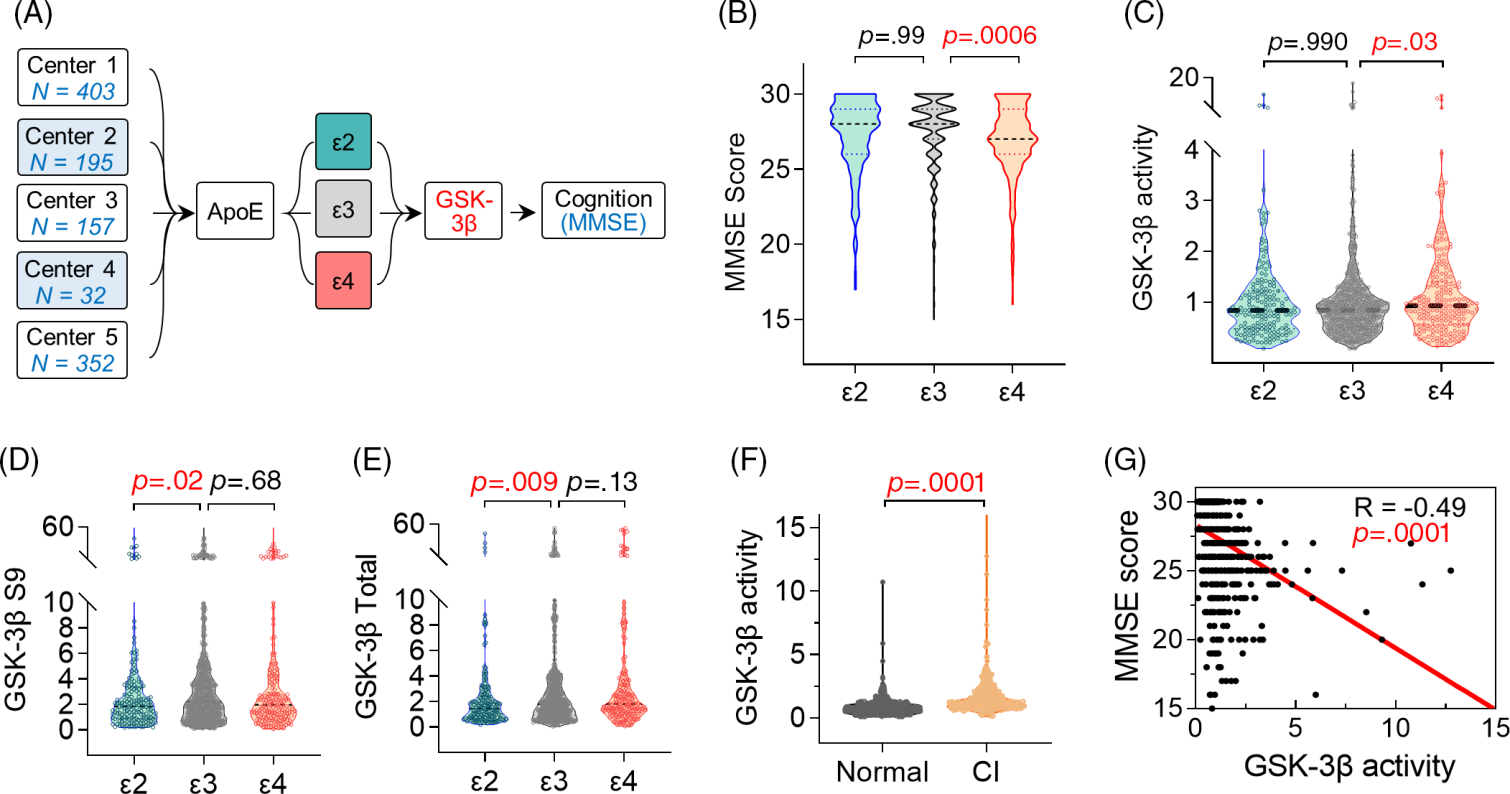
*ApoE ε4*‐carriers show poorer cognitive performance and an elevated GSK‐3β activity. (A) Study design. (B, C) The ε4 group had lower MMSE scores (B) and higher GSK‐3β activity (C) compared with ε3 control. (D, E) The ε4 group showed comparable level of S9 and total GSK‐3β to the ε3, and both S9 and total GSK‐3β in the ε2 were significantly decreased compared with ε3. (B–E) One‐way ANOVA followed by Kruskal–Wallis multiple comparisons test. (F) T2DM patients with cognitive impairment (CI) showed significant increased GSK‐3β activity compared with the T2DM with normal cognitive function. Mann–Whitney test for nonnormally distributed data. (G) GSK‐3β activity was negatively correlated with MMSE scores in T2DM patients. Spearman correlation analysis. N = 1139. ANOVA, analyis of variance; ApoE, apolipoprotein E; CI, cognitive impairment; GSK‐3β, glycogen synthase kinase‐3 beta; MMSE, Mini‐Mental State Examination; T2DM, type 2 diabetes mellitus.

A total of 1139 T2DM patients were recruited from five medical centers in Wuhan, China, between January 2012 and November 2018 (NCT01830998, Clinical Trials.gov), including 403 patients from the Central Hospital of Wuhan, 195 from Wuhan No. 1 Hospital, 157 from Wuhan General Hospital of Guangzhou Military Region, 32 from Liyuan Hospital of Tongji Medical College of HUST and 352 from Jianghan Road Community Hospital (Figure 1A).

The T2DM patients were diagnosed by the World Health Organization National Diabetic Group Criteria of 2006.^15^ The inclusion and exclusion criteria in the cohort were as presented previously.^12^ Briefly, the inclusion criteria include (1) age ≥45 years, (2) long‐term residence (≥5 years) (3) having ability to complete the neuropsychological test and write informed consent. The exclusion criteria are as follows: (1) having dementia before T2DM; (2) having history of either head trauma, stroke, brain tumor, coma, transient ischemic attack, epilepsy, or other central nervous system diseases that could cause dementia; (3) having auditory/visual disorders, thyroid disease, history of using possible or known drugs affecting cognitive function abuse, alcohol addiction, diagnosed depression, schizophrenia, and other psychiatric disorders such as acute stress disorder, posttraumatic stress disorder, acute transient psychosis, and so on.

The study protocol was approved by Tongji Medical School Ethics Committee in accordance with the principles of the Helsinki Declaration II. All participants have provided written informed consent.

### 2.2 Study procedures

All participants received a series of complete assessments including neuropsychological evaluation, conventional medical history and physical examinations. The neuropsychological evaluations included the Mini‐Mental State Examination (MMSE) and the Clinical Dementia Rating, both of which were performed by experts who had received neurology training or was working in department of neurology/endocrine. Cognitive impairment was diagnosed based on Petersen's MCI criteria: memory complaint, normal activities of daily living, normal general cognitive function, abnormal memory for age, and MMSE scores between 24 and 26. Besides, patients with MMSE scores between 10 and 23 were diagnosed with moderate dementia.

All blood samples were collected and immediately separated into platelet, white blood cells, red blood cells, and plasma through centrifugation within 2 hours. The aliquots were stored at −80°C. DNA was prepared from white blood cells and the ApoE genotyping was performed by polymerase chain reaction (PCR) using Gerard's method with modifications in the multiplex amplification refractory mutation system.^16^ Total GSK‐3β and inactive GSK‐3β (pS9) in platelet were measured by dot blot as previously described,^12^ using antibodies of GSK‐3β‐total (Cell Signaling, 12456, 1:1000) and GSK‐3β‐S9 (Cell Signaling, 5558, 1:1000). Data were normalized by the same control to adjust the influence of different detection periods on the results. The ratio of GSK‐3β‐total/S9 was calculated to indicate the GSK‐3β activity.^12^ Glycated hemoglobin (HbA1c) and fasting plasma glucose (FPG) was detected through venipuncture in the morning before breakfast.

### 2.3 Statistical analysis

All data were analyzed using SPSS 26.0 IBM software and Prism 8.0 (GraphPad). The variables were presented as mean ± SD, median (interquartile range) or frequency (%). Group differences in MMSE score, platelet GSK‐3β, and other basic characteristics were assessed using analysis of variance (ANOVA) followed by the Tukey's multiple comparisons test (normal distribution), Kruskal–Wallis test (skewed distribution), or χ^2^ test (categorical variables). *p* value <.05 was considered as statistically significant.

Multivariable logistic regression analysis was performed to evaluate the independent association between ApoE haplotype and cognitive impairment (yes/no) among T2DM patients. The crude analysis included only ApoE polymorphism without any adjustment (model 1). Adjusted analyses were also performed by adjustment for age and sex (model 2) or age, sex, diabetes duration, diabetic complications, HbA 1c, FPG, history of hypertension (yes/no), hyperlipidemia (yes/no), and coronary heart disease (CHD) (yes/no) (model 3).

Multivariate linear regression was used to evaluate the associations between ApoE haplotypes with MMSE scores and GSK‐3β, respectively, with adjustment for the preceding variables in independent models. Spearman correlation analyses were performed between GSK‐3β with variables (age, MMSE, diabetes duration, HbA1c, FPG).

Mediation analyses were used based on the previous method^17^ to explore the correlations among ApoE4, MMSE scores, and GSK‐3β. The interpretation of mediation analyses results was referred to related literatures.^18^

## 3 RESULTS

### 3.1 Demographic and clinical characteristics

Data of 1139 T2DM patients from five medical centers were included in the cohort (Figure 1A). Their average age was 64.8 (SD = 8.0, N = 645 for age ≤65, N = 494 for age >65). Overall *ApoE* allele frequencies were 0.095 for ε2, 0.815 for ε3, and 0.09 for ε4. Overall haplotype frequencies were 0.007 for ε2/ε2, 0.143 for ε2/ε3, 0.022 for ε2/ε4, 0.678 for ε3/ε3, 0.131 for ε3/ε4, and 0.019 for ε4/ε4 (Table 1). In consistent with previous studies,^4^ allele ε3 and genotype ε3/ε3 were most common in the cohort (Table 1). In the present study, all patients were grouped as ε2 (ε2/ε2, ε2/ε3 genotypes), ε3 (ε3/ε3 genotype), and ε4 (ε2/ε4, ε3/ε4, ε4/ε4 genotypes) carriers using a codominant Mendelian inheritance model. Data from ε3 carriers were used as control in the present study. Demographic and clinical characteristics of the cohort were summarized in Table 2. No statistical differences among three groups were observed in age, sex, diabetes duration, HbA 1c, FPG, hypertension, hyperlipidemia, diabetic complications and CHD (Table 2).

**TABLE 1:** Frequency distribution of different ApoE genotypes.

ApoE Genotypes	Male	Female	Overall	
	N	N	N	Frequency (%)
ε2/ε2	1	7	8	0.7
ε2/ε3	63	100	163	14.3
ε3/ε3	315	457	772	67.8
ε2/ε4	6	19	25	2.2
ε3/ε4	72	77	149	13.1
ε4/ε4	8	14	22	1.9
Total	465	674	1139	100

Abbreviation: ApoE, apolipoprotein E.

**TABLE 2:** Group characteristics of different ApoE genotypes.

	ε2	ε3	ε4	*p* value
N	171	772	196	‐
Age (years)	65.9 (8.6)	64.5 (7.9)	65.2 (7.9)	.067
Sex (male, %)	64 (37.4)	315 (40.6)	86 (43.9)	.455
Hypertension (yes, %)	96 (56.1)	449 (58.1)	97 (49.5)	.092
Hyperlipemia (yes, %)	35 (20.5)	146 (18.9)	38 (19.4)	.895
CHD (yes, %)	17 (10.0)	75 (9.7)	18 (9.2)	.966
DC (yes, %)	67 (39.2)	355 (43.4)	95 (48.5)	.172
HbA1c (%Hb)	7.87 (2.18)	7.96 (1.90)	8.05 (1.92)	.736
FPG (mg/dl)	8.60 (3.16)	9.16 (3.74)	8.89 (3.60)	.177
DD (years)	5.5 (2.3.10)	7.0 (2.12)	6.0 (2.12)	.316
MMSE	28 (26.29)	28 (27.29)	27 (26.29)	.001
CN (MMSE ≥27, yes, %)	121 (70.8)	586 (75.9)	128 (65.3)	.008
CI (MMSE<27, yes, %)	50 (29.2)	186 (24.1)	68 (34.7)	.008
MCI (24 ≤ MMSE≤26, yes, %)	35 (20.5)	126 (16.3)	52 (26.5)	.004
MD (10 ≤ MMSE≤23, yes, %)	15 (8.8)	60 (7.8)	16 (8.1)	.905

*Note*: All data were presented as mean (SD) or percentage except for DD and MMSE, which were presented as medians (interquartile range). *p* values were calculated by ANOVA or chi‐square tests.

Abbreviations: ANOVA, analysis of variance; ApoE, apolipoprotein E; CHD, coronary heart disease; CI, cognitive impairment; CN, cognitive normal; DC, diabetic complications; DD, diabetes duration; FPG, fasting plasma glucose; HbA1c, hemoglobin A1c; MCI, mild cognitive impairment; MD, moderate dementia; MMSE, Mini‐Mental State Examination.

### 3.2 ApoE ε4 is associated with cognitive impairment in T2DM patients

We found that the carriers of allele ε4 (*p* = .0006) but not ε2 (*p* = .99) showed a significantly reduced MMSE score when using ε3 as the reference (Figure 1B). Following the stratification of sex and age, two major interfering factors of cognitive performance,^19^, ^20^ we found that female (*p* = .0007) but not male (*p* = .39) ε4 carriers had lower MMSE scores than their ε3 counterparts (Figure S1A), indicating female patients may be more vulnerable than male patients to ApoE ε4 induced cognitive impairment. Simultaneously, midlife (age ≤65 years, *p* = .008) ε4 carriers showed significantly worse cognitive performance, and late‐life (age >65 years, *p* = .21) ε4 carriers showed an decrease tendency of MMSE scores but no statistical significance (Figure S1B).

We next performed logistic analyses to evaluate whether the *ApoE* polymorphism is independently associated with cognitive impairment in T2DM patients. We found that ε4 (odds ratio [OR] = 1.610 [1.156, 2.241], *p* = .005) but not ε2 (OR = 1.129 [0.795, 1.603], *p* = .499) showed significant association with higher risk of cognitive impairment compared with ε3 (Table 3, model 1). The associations were still significant following adjustments by age and sex (OR = 1.618 [1.140, 2.296], *p* = .007, Table 3, model 2), or by age, sex, diabetes duration, HbA1c, FPG, history of hypertension, hyperlipidemia, CHD, and diabetic complications (OR = 1.733 [1.142, 2.628], *p* = .010, Table 3, model 3).

**TABLE 3:** Logistic regression analyses of the association between *ApoE* polymorphisms and the risk of cognitive impairment.

	Variables	β	SE	*p* value	OR (95% CI)
Model 1	ε2	0.121	0.179	.499	1.129 (0.795–1.603)
	**ε4**	**0.476**	**0.169**	**.005**	**1.610 (1.156–2.241)**
Model 2	ε2	−0.017	0.191	.929	0.983 (0.676–1.430)
	**ε4**	**0.481**	**0.179**	**.007**	**1.618 (1.140–2.296)**
Model 3	ε2	−0.111	0.232	.633	0.895 (0.569–1.410)
	**ε4**	**0.550**	**0.213**	**.010**	**1.733 (1.142–2.628)**

*Note*: ε3 was used as the reference. Model 1, without adjustment. Model 2, adjusted for age and sex. Model 3, adjusted for age, sex, hypertension (Yes/No), hyperlipemia (Yes/No), CHD (Yes/No), diabetic complications (Yes/No), HbA1c, FPG, and diabetes duration.

Abbreviations: ApoE, apolipoprotein E; CHD, coronary heart disease; FPG, fasting plasma glucose; HbA1c, hemoglobin A1c; OR, odds ratio.

Additionally, significant association of cognitive impairment with age and FPG level were also detected by logistic regression analysis (Table S1) and multivariate linear regression analysis (Table S2), respectively.

### 3.3 Platelet GSK‐3β activation is independently associated with ApoE ε4 and cognitive impairment

Consistent with our previous findings that T2DM patients with MCI showed higher *ApoE **ε**4*‐carrying frequency and elevated GSK‐3β activity compared with those without cognitive impairments,^12^ we found here that **ε**4 (*p* = .03) but not **ε**2 carriers (*p* = .99) exhibited significantly increased GSK‐3β activity compared with the **ε**3 group (Figure 1C). However, though T2DM patients also showed overall gender and age differences in GSK‐3β activity (Figure S2A, C), these differences were no longer significant following the stratification by *ApoE* genotypes (Figure S2B, D).

In addition, it should be noted that ε2 carriers showed statistically lower levels of both total and pS9 GSK‐3β, (*p* = .009; *p* = .02) but their ratio (GSK‐3β activity, *p* = .99) remained unchanged compared with **ε**3 (Figure 1D, E), suggesting an overall reduction of GSK‐3β in ε2‐carried T2DM patients. In the present study, we mainly focused on the association between **ε**4 and GSK‐3β.

We next analyzed the correlation between GSK‐3β activity and *ApoE* polymorphism. Activation of GSK‐3β was significant correlated with ε4 (*p* < .05) but not ε2 (*p* > .05) in linear regression analysis without (Table 4,model 1) or with (Table 4, model 2 and 3) adjustment by age, sex, diabetes duration, HbA1c, FPG, history of hypertension, hyperlipidemia, CHD, and diabetic complications. These data indicate that *ApoE ε4* genotype is independently associated with an increased GSK‐3β activity in T2DM patients.

**TABLE 4:** Linear regression analyses of association between *ApoE* polymorphisms with GSK‐3β activity.

	Variables	β	SE	95% CI	*p* value
Model 1	ε2	0.027	0.088	−0.146 to 0.200	.759
	**ε4**	**0.188**	**0.087**	**0.018 to 0.359**	**.031**
Model 2	ε2	−0.002	0.087	−0.173 to 0.170	.984
	**ε4**	**0.180**	**0.086**	**0.012 to 0.349**	**.036**
Model 3	ε2	0.040	0.094	−0.143 to 0.224	.891
	**ε4**	**0.235**	**0.094**	**0.051 to 0.419**	**.007**

*Note*: ε3 was used as the reference. Model 1, without adjustment. Model 2, adjusted for age and sex. Model 3, adjusted for age, sex, hypertension (Yes/No), hyperlipemia (Yes/No), CHD (Yes/No), diabetic complications (Yes/No), HbA1c, FPG, and diabetes duration.

Abbreviations: ApoE, apolipoprotein E; CHD, coronary heart disease; FPG, fasting plasma glucose; GSK‐3β, glycogen synthase kinase‐3 beta; HbA1c, hemoglobin A1c.

We also found in the current cohort that T2DM patients with cognitive impairment (CI) showed elevated GSK‐3β activity compared with those with normal cognition (*p* < .0001, Figure 1F). Meanwhile, GSK‐3β activity was negatively correlated with MMSE scores (R = −0.49, *p* < .0001, Figure 1G), and positively correlated with diabetes duration, HbA1c, and FPG level (Table S3). The consistent results were observed in regression analysis adjustment by age, sex, *ApoE ε4*, HbA1c, FPG and diabetes duration, history of hypertension, hyperlipemia, CHD, and diabetic complications (Table 5 and Table S4).

**TABLE 5:** Logistic regression analyses of the association between GSK‐3β activity and the risk of cognitive impairment.

	Variables	β	SE	*p* value	OR (95% CI)
Model 1	GSK3βT/S9	1.283	0.169	<.001	3.606 (2.590 –5.020)
Model 2	GSK3βT/S9	1.263	0.171	<.001	3.538 (2.531 –4.944)
Model 3	GSK3βT/S9	1.29	0.181	<.001	3.633 (2.546 –5.186)

*Note*: Model 1, without adjustment. Model 2, adjusted for age and sex. Model 3, adjusted for age, sex, *ApoE* ε4 (Yes/No), hypertension (Yes/No), hyperlipemia (Yes/No), CHD (Yes/No), diabetic complications (Yes/No), HbA1c, FPG, and diabetes duration. GSK3βT/S9 represents GSK‐3β activity.

Abbreviations: ApoE, apolipoprotein E; CHD, coronary heart disease; FPG, fasting plasma glucose; GSK‐3β, glycogen synthase kinase‐3 beta; HbA1c, hemoglobin A1c.

### 3.4 GSK‐3β activation mediates the ApoE ε4‐associated cognitive impairment in T2DM

**FIGURE 2:**
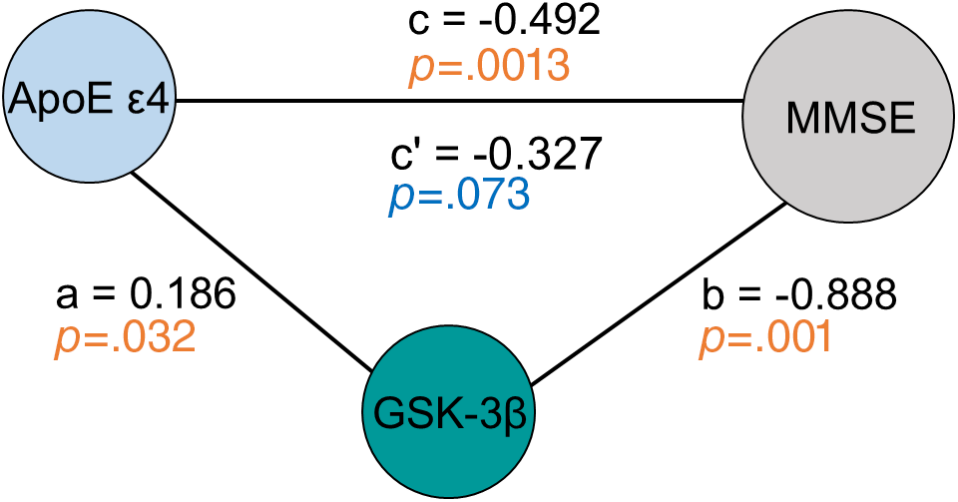
GSK‐3β activation mediates the association of *ApoE ε4* and cognitive impairment in T2DM patients. The direct pairwise paths among ApoE ε4, GSK‐3β activity, and MMSE scores analyzed by Baron and Kenny's mediation analysis. The a, b and c were regression coefficients indicating the strength of each association. The c' represented the regression coefficient of direct path between *ApoE ε4* and MMSE score. The statistical difference of direct *ApoE ε4*‐MMSE path was nonsignificant (*p* = .073) when adjusted by GSK‐3β activity. N = 1139. ApoE, apolipoprotein E; GSK‐3β, glycogen synthase kinase‐3 beta; MMSE, Mini‐Mental State Examination; T2DM, type 2 diabetes mellitus.

Previous studies have shown that *ApoE ε4* is an upstream regulator of GSK‐3β activity in tauopathy and AD.^13^, ^14^ We wonder whether the cognitive decline in *ApoE ε4*‐carried T2DM patients is mediated by the upregulation of GSK‐3β activity. We found using Baron and Kenny's mediation analyses that the regression coefficient of direct path between *ApoE ε4* and cognitive performance was no longer significant when adjusted by GSK‐3β activity (c = −0.492, *P* = .0013, and c′ = −0.327, *p* = .073 in Figure 2). These data indicate that GSK‐3β activation plays as a mediator between *ApoE ε4* and cognitive impairment in T2DM.

## 4 DISCUSSION

Human ApoE is an ~34‐kDa protein consisting of 299 amino acids that transports cholesterol and other lipids in the plasma and the central nervous system (CNS) by binding to cell surface ApoE receptors.^21^ It plays important roles in cell proliferation, plasma membrane repair, remyelination of new axons,^22^ modulation of glutamate receptor function, and synaptic plasticity,^23^ and as well as maintenance of blood–brain barrier integrity.^24^ Among the three *ApoE* allelic variants, *ApoE ε4* is the strongest genetic risk factor for late‐onset AD. The ε4‐carried AD patients generally show advanced onset and worse cognitive performance relative to ε3 carriers.^5^ By contrast, ε2 seems to play a neural protective role in AD.^19^ However, the association of *ApoE* gene polymorphism with cognitive functions in T2DM patients has been elusive. Several studies demonstrated that only ε4 was associated with lower cognitive scores in T2DM cohort.^25^, ^26^ We also observed in the current cohort that the ε4 carriers showed significant association with low MMSE score, that is, high risk of cognitive impairment in T2DM patients.

Although our results indicated nonsignificant associations of *ApoE ε2* with both cognitive function and GSK‐3β in the current cohort, it seems not entirely uninvolved. Some other studies found that ε2 was also a risk factor of cognitive decline in T2DM^4^, ^7^, ^8^; consistently, *ApoE ε2*‐carriers showed increased risk of cerebral amyloid angiopathy, a cerebrovascular disorder caused by deposition of amyloid Aβ aggregates and correlates with presenile cognitive decline.^27^ The inconsistencies remaining in the influence of *ApoE ε2* on cognition in T2DM patients might be attributed to racial, ethical, or regional differences, which deserves further investigation.

*ApoE ε4* could exert influence on cognition through various ways. Take AD for example: it can promote Aβ aggregation and simultaneously inhibit Aβ clearance,^28^, ^29^, ^30^ trigger synaptic dysfunction, disrupt the blood–brain barrier, exaggerate tauopathy‐mediated neurodegeneration, and dysregulate insulin signaling.^13^, ^31^, ^32^ However, direct evidence illustrating how these factors are affected by *ApoE ε4* in T2DM is still lacking. We revealed in the present study through correlation and mediation analyses that *ApoE ε4* can lead to cognitive impairments in T2DM patients by elevating GSK‐3β activity.

Indeed, in vitro studies showed that *ApoE4* expression can increase both GSK‐3β expression and its activity.^14^, ^33^ Consistently, we unveiled in the present study that ε4‐carried T2DM patients had higher GSK‐3β activity and poorer cognitive performance. Meanwhile, the *ApoE* gene polymorphism, GSK‐3β, and MMSE score showed significant pairwise associations in regression analysis. Importantly, in the mediation analyses, GSK‐3β activity appeared to be a complete mediative factor between *ApoE ε4* and cognitive performance in T2DM patients. To our best knowledge, this is the first report evidencing the relationships of *ApoE* gene polymorphisms, GSK‐3β activity, and the cognitive functions in T2DM patients.

**FIGURE 3:**
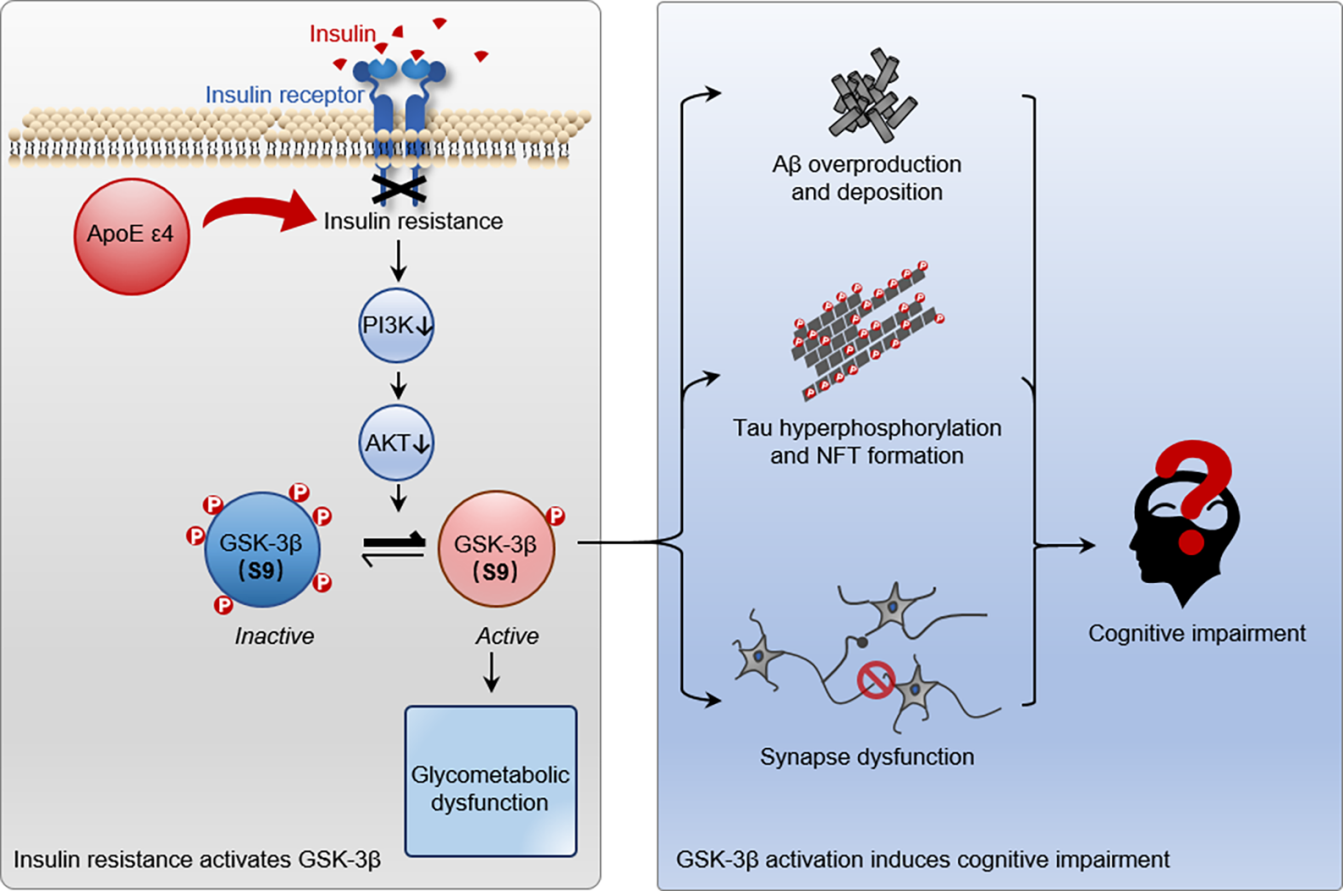
Working model. Insulin resistance in T2DM patients can upregulate GSK‐3β by inhibiting PI3K‐Akt cascade and S9‐phosphorylation of GSK‐3β (left). Activation of GSK‐3β deregulates glycometabolism and induces multiple AD‐like pathologies such as β‐amyloid overproduction and accumulation, tau hyperphosphorylation, and neurofibrillary tangle formation, synapse dysfunction, leading to neurodegeneration and cognitive impairments (right). In summary, GSK‐3β activation mediates the *ApoE ε4*‐associated cognitive impairments in T2DM. AD, Alzheimer disease; ApoE, apolipoprotein E; GSK‐3β, glycogen synthase kinase‐3 beta; MMSE, Mini‐Mental State Examination; NFT, neurofibrillary tangle; T2DM, type 2 diabetes mellitus.

Elevated GSK‐3β activity has been implicated in cognitive dysfunctions associated with various neurodegenerative disorders.^12^, ^34^ In T2DM, the activity of Akt, an upstream serine/threonine kinase of GSK‐3β that plays pivotal roles in cell metabolism, is generally inhibited as a result of insulin resistance, which is the main cause of glucose metabolism dysfunction, lipid accumulation, and protein synthesis inhibition in patients.^3^, ^35^ Thereby, GSK‐3β is disinhibited and overactivated and thus lead to downstream pathologies such as tau hyperphosphorylation^36^ and Aβ toxicity^37^ (Figure 3). Inhibition of GSK‐3β in diabetic mice was effective in decreasing body weight, downregulating serum glucose levels, increasing serum insulin, and improving cognitive functions.^38^ Importantly, *ApoE ε4* expression can exacerbate the insulin resistance and hinder both cerebral and peripheral responses to insulin signaling,^26^, ^39^ possibly by trapping insulin receptor in the endosomes.^13^ We found here that ε4‐carried T2DM patients showed elevated GSK‐3β activity in periphery blood, which was positively correlated with diabetes duration, plasma HbA1c, and FPG level, which provided novel information to link ApoE gene polymorphisms and GSK‐3β with cognitive functions in T2DM patients. We measured platelet GSK‐3β because we think platelets contain more abundant information related to CNS and are more stable than serum as well as other types of blood cells. Many evidences have suggested that platelets, the fragments shed by megakaryocytes, have many biological similarities with neurons.^40^ Platelets, the peripheral synaptic vesicles, also share many of the same secretory pathways and transporters as the synaptic terminals of neurons during neurotransmitter uptake and packaging.^41^ We have also reported that the platelet GSK‐3β activity could be a peripheral biomarker for MCI, because it showed increase in T2DM with MCI (T2DM‐MCI) patients compared to T2DM without MCI (T2DM‐nMCI).^12^, ^42^, ^43^, ^44^ However, how peripheral insulin homeostasis influence CNS is an important future direction.^45^, ^46^

GSK‐3β inhibitors hold therapeutic potentials for T2DM and AD possibly by preventing the aggregation of β‐amyloid (Aβ), inhibiting the hyperphosphorylation of tau protein, or reducing neuroinflammation in animal models,^47^, ^48^, ^49^, ^50^ but their effectiveness for improving cognitive impairment need further proof. In addition, GSK‐3β inhibitors were also effective in suppressing malignant cell proliferation,^51^ so many of them have also entered clinic trials for the treatment of advanced cancers (https://clinicaltrials.gov/ct2/show/NCT03678883?term=9-ing&rank=1).

It should be noted that the cognitive impairment in the current cohort was observed only in female and midlife (≤65 years old) ε4‐carried patients. Partially inconsistently, elevated GSK‐3β activity was found in female but late‐life (>65 years old), and these differences even turned nonsignificant when data were stratified by *ApoE* genotypes.

The age‐ and gender‐dependent impacts of *ApoE* alleles on cognitive function could be attributed to various mechanisms,^52^, ^53^ such as physiological and neural development,^54^ hormone levels,^55^ and lifestyle factors.^56^ Understanding the multiple mechanisms through which age and gender interact with ApoE alleles to influence cognitive function is crucial for developing personalized interventions and treatments for cognitive decline. Nevertheless, there are several limitations of the present study. First, we used here a cross‐sectional study design, so that the causal inference should be cautious. Moreover, residual confounders and potential bias cannot be completely addressed due to the nature of the observational study. Second, this study included only the Han population in Wuhan, and generalization is limited. Considering the sample capacity and eternities disparities, findings should be replicated in other ethnic and racial groups.

In summary, our results reveal that *ApoE ε4* genotype was associated with cognitive dificits in Chinese Han T2DM patients, and GSK‐3β plays a mediative role between *ApoE ε4* and cognitive impairment. The glycometabolic dysregulation and the AD‐like Aβ and tau pathology induced by GSK‐3β are presumably involved in the cognitive deterioration. Future longitudinal follow‐up in combination with cerebrospinal fluid or brain tissue sample, and as well as in vitro and animal studies will reveal the causal role of *ApoE ε4*, GSK‐3β and the cognitive functions in T2DM patients.

## AUTHOR CONTRIBUTIONS

Jian‐Zhi Wang and Yang Gao designed this research; Zhipeng Xu and Yanchao Liu made major contributions in organizing this project; Zhipeng Xu, Yanchao Liu, Benrong He, Haitao Yu, Yao Zhang, Yuying Wang, and Yi Liang recruited the patients, performed cognitive function tests, and collected blood samples. Zhipeng Xu Yanchao Liu, Benrong He, and Yang Gao separated and detected blood samples. Yang Gao, J. Z. and Haitao Yu performed the statistical analysis and data interpretation. Yang Gao, Jian‐Zhi Wang, and J. Z. wrote the manuscript. All authors read and approved the final manuscript.Jian‐Zhi Wang is the supervisor of this work who has full access to all the data in the study and take responsibility for the data integrity and accuracy.

## FUNDING INFORMATION

This study was supported in parts by the Natural Science Foundation of China (82230041, 91949205, 31730035, 81721005), the National Key R&D Program of China (2016YFC1305800), the Fundamental Research Funds for the Central Universities (YCJJ202203019), the Hubei Province scientific research project (WJ2021M041), the Wuhan Health Science Foundation (WX20Q04), the Guangdong Provincial Key S&T Program (018B030336001). The funders neither played a role in the study design, conduct, data collection, analysis, interpretation, nor participated in the preparation, review, or approval of the manuscript.

## CONFLICT OF INTEREST STATEMENT

The authors have no conflict of interest to declare.

## Supporting information


**Figure S1.** *ApoE ε4*‐carried type 2 diabetes mellitus (T2DM) patients show gender and age difference in cognitive performance. (A) Female but not male ε4 carriers had lower Mini‐Mental State Examination (MMSE) score than the ε3 counterparts. (B) Merely midlife (≤65 years old) but not late‐life (>65 years old) patients showed decreased MMSE score compared with ε3 group. One‐way analysis of variance followed by Kruskal–Wallis multiple comparisons test.
**Figure S2.** T2DM patients show gender and age difference in GSK‐3β activity. (A) Female patients exhibited higher GSK‐3β activity than males. (B) Patients with different ApoE genotypes showed no statistical difference in GSK‐3β activity both for females and males. (C) Late‐life patients exhibited higher GSK‐3β activity than the midlife group. (D) Patients with different ApoE genotypes showed no statistical difference in GSK‐3β activity both for midlife and late‐life patients. A, C, Mann–Whitney test for nonnormally distributed data; B, D, One‐way analysis of variance followed by Kruskal–Wallis multiple comparisons test.
**Table S1.** Logistic regression analysis of association between potential risk factors and cognitive impairment. ε3 was used as the reference. Analyses were performed with adjustment for age, sex, hypertension (Yes/No), hyperlipemia (Yes/No), CHD (Yes/No), diabetic complications (Yes/No), HbA1c, FPG, and diabetes duration. ε3 was used as the reference. CHD, coronary heart disease; DC, diabetic complications; DD, diabetes duration; HbA1c, hemoglobin A1c; FPG, fasting plasma glucose.
**Table S2.** Linear regression analysis of association between potential risk factors with cognitive performance. ε3 was used as the reference. Analyses were performed with adjustment for age, sex, hypertension (Yes/No), hyperlipemia (Yes/No), CHD (Yes/No), diabetic complications (Yes/No), HbA1c, FPG, and diabetes duration. ε3 was used as the reference. CHD, coronary heart disease; DC, diabetic complications; DD, diabetes duration; HbA1c, hemoglobin A1c; FPG, fasting plasma glucose.
**Table S3.** Correlation analysis of glycogen synthase kinase‐3β (GSK‐3β) with clinical parameters. DD, diabetes duration; FPG, fasting plasma glucose; HbA1c, hemoglobin A1c; MMSE, Mini‐Mental State Examination.
**Table S4.** Linear regression analysis of association between other factors with glycogen synthase kinase‐3β (GSK‐3β) activity. ε3 was used as the reference. Analyses were performed with adjustment for age, sex, hypertension (Yes/No), hyperlipemia (Yes/No), CHD (Yes/No), diabetic complications (Yes/No), HbA1c, FPG, diabetes duration. ε3 was used as the reference. CHD, coronary heart disease; DC, diabetic complications; DD, diabetes duration; HbA1c, hemoglobin A1c; FPG, fasting plasma glucose.

## Data Availability

All data are available in the manuscript or the supplementary materials. Other data supporting the findings of this study are available from the leading corresponding author, prof. Jian‐Zhi Wang (wangjz@mail.hust.edu.cn), upon reasonable request.
