# Supplementary material for: GSK‐3β activation mediates apolipoprotein E4‐associated cognitive impairment in type 2 diabetes mellitus: A multicenter, cross‐sectional study
Source: J Diabetes. 2023 Sep 12;16(1):e13470. doi: 10.1111/1753-0407.13470 (PMC10809305; doi:10.1111/1753-0407.13470)
Supplement: Supplementary file 1 — Figure S1. ApoE ε4‐carried type 2 diabetes mellitus (T2DM) patients show gender and age difference in cognitive performance. (A) Female but not male ε4 carriers had lower Mini‐Mental State Examination (MMSE) score than the ε3 counterparts. (B) Merely midlife (≤65 years old) but not late‐life (>65 years old) patients showed decreased MMSE score compared with ε3 group. One‐way analysis of variance followed by Kruskal–Wallis multiple comparisons test. Figure S2. T2DM patients show gender and age difference in GSK‐3β activity. (A) Female patients exhibited higher GSK‐3β activity than males. (B) Patients with different ApoE genotypes showed no statistical difference in GSK‐3β activity both for females and males. (C) Late‐life patients exhibited higher GSK‐3β activity than the midlife group. (D) Patients with different ApoE genotypes showed no statistical difference in GSK‐3β activity both for midlife and late‐life patients. A, C, Mann–Whitney test for nonnormally distributed data; B, D, One‐way analysis of variance followed by Kruskal–Wallis multiple comparisons test. Table S1. Logistic regression analysis of association between potential risk factors and cognitive impairment. ε3 was used as the reference. Analyses were performed with adjustment for age, sex, hypertension (Yes/No), hyperlipemia (Yes/No), CHD (Yes/No), diabetic complications (Yes/No), HbA1c, FPG, and diabetes duration. ε3 was used as the reference. CHD, coronary heart disease; DC, diabetic complications; DD, diabetes duration; HbA1c, hemoglobin A1c; FPG, fasting plasma glucose. Table S2. Linear regression analysis of association between potential risk factors with cognitive performance. ε3 was used as the reference. Analyses were performed with adjustment for age, sex, hypertension (Yes/No), hyperlipemia (Yes/No), CHD (Yes/No), diabetic complications (Yes/No), HbA1c, FPG, and diabetes duration. ε3 was used as the reference. CHD, coronary heart disease; DC, diabetic complications; DD, diabetes duration; Hb [file JDB-16-e13470-s001.docx]

**Supplementary materials**


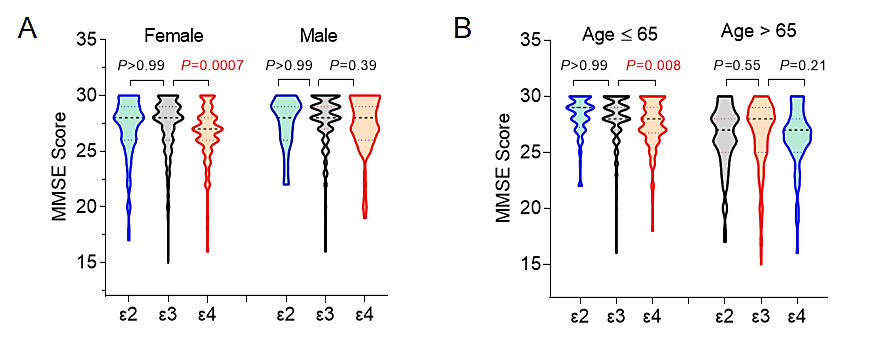


**Figure S1. *ApoE ε4*-carried T2DM patients show gender and age difference in cognitive performance.**

(**A**) Female but not male ε4 carriers had lower MMSE score than the ε3 counterparts. (**B**) Merely midlife (≤65 years old) but not late-life (>65 years old) patients showed decreased MMSE score compared with ε3 group. One-way ANOVA followed by Kruskal-Wallis multiple comparisons test.


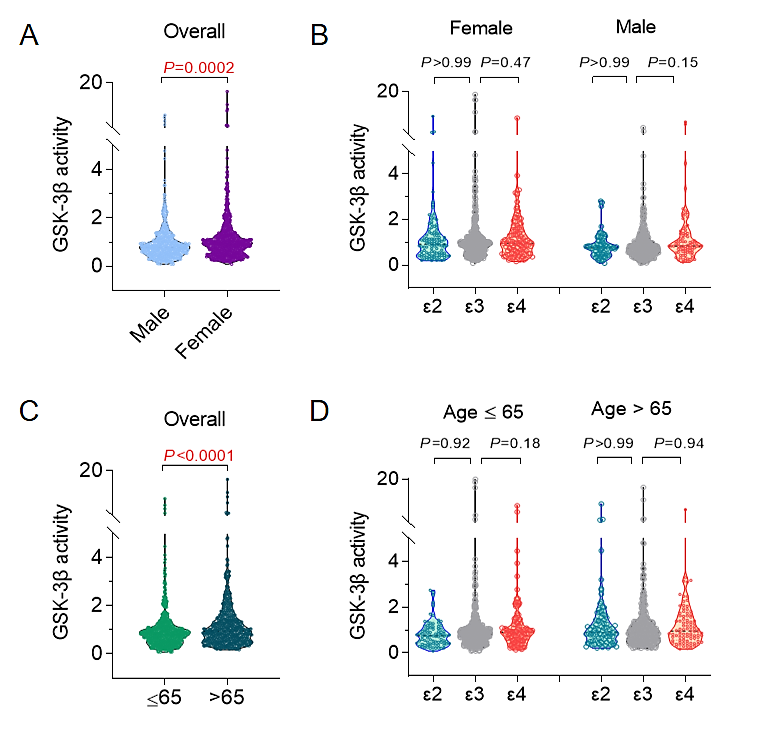
**Figure S2. T2DM patients show gender and age difference in GSK-3β activity.**

(**A**) Female patients exhibited higher GSK-3β activity than males. (**B**) Patients with different ApoE genotypes showed no statistical difference in GSK-3β activity both for females and males. (**C**) Late-life patients exhibited higher GSK-3β activity than the mildlife group. (**D**) Patients with different ApoE genotypes showed no statistical difference in GSK-3β activity both for midlife and late-life patients. A, C, Mann-Whitney test for non‐normally distributed data; B, D, One-way ANOVA followed by Kruskal-Wallis multiple comparisons test.

**Table S1. logistic regression analysis of association between potential risk factors and cognitive impairment.**

| Variables | β | SE | *P* value | OR (95%CI) |
| --- | --- | --- | --- | --- |
| ε2 | -0.111 | 0.232 | 0.633 | 0.895 (0.569,1.410) |
| **ε4** | 0.550 | 0.213 | **0.010** | **1.733 (1.142,2,628)** |
| **Age** | 0.086 | 0.012 | **0.000** | **1.089 (1.064,1.115)** |
| Sex | -0.237 | 0.178 | 0.185 | 0.789 (0.556,1.120) |
| Hypertension | -0.157 | 0.178 | 0.378 | 0.855 (0.603,1.212) |
| Hyperlipidemia | 0.262 | 0.210 | 0.212 | 1.299 (0.862,1.959) |
| CHD | 0.180 | 0.296 | 0.544 | 1.197 (0.670,2.138) |
| DC | -0.162 | 0.181 | 0.371 | 0.850 (0.596,1.213) |
| DD | 0.005 | 0.013 | 0.705 | 1.005 (0.979,1.032) |
| HbA 1c | 0.040 | 0.050 | 0.429 | 1.040 (0.943,1.148) |
| **FPG** | 0.089 | 0.026 | **0.001** | **1.093 (1.038,1.151)** |

*Notes*: ε3 was used as the reference. Analyses were performed with adjustment for age, sex, hypertension (Yes / No), hyperlipemia (Yes / No), CHD (Yes / No), diabetic complications (Yes / No), HbA1c, FPG and diabetes duration. ε3 was used as the reference. Abbreviations: CHD, coronary heart disease; DC, diabetic complications; DD, diabetes duration; HbA1c, hemoglobin A1c; FPG, fasting plasma glucose.

**Table S2. Linear regression analysis of association between potential risk factors with cognitive performance**

| Variables | β | SE | 95% CI | *P* value |
| --- | --- | --- | --- | --- |
| ε2 | 0.232 | 0.190 | -0.141, 0.606 | 0.223 |
| **ε4** | **-0.596** | 0.190 | -0.970, -0.223 | **0.002** |
| **Age** | **-0.087** | 0.010 | -0.106, -0.067 | **0.000** |
| Sex | 0.201 | 0.146 | -0.086, -0.488 | 0.169 |
| Hypertension | -0.033 | 0.148 | -0.324, 0.258 | 0.823 |
| Hyperlipidemia | -0.286 | 0.180 | -0.640, 0.067 | 0.112 |
| CHD | 0.096 | 0.264 | -0.422, 0.614 | 0.716 |
| DC | 0.250 | 0.150 | -0.044, 0.544 | 0.096 |
| DD | -0.008 | 0.012 | -0.031, 0.016 | 0.518 |
| HbA 1c | 0.014 | 0.042 | -0.069, 0.097 | 0.739 |
| **FPG** | **-0.070** | 0.023 | -0.116, -0.025 | **0.003** |

*Notes*: ε3 was used as the reference. Analyses were performed with adjustment for age, sex, hypertension (Yes / No), hyperlipemia (Yes / No), CHD (Yes / No), diabetic complications (Yes / No), HbA1c, FPG and diabetes duration. ε3 was used as the reference. Abbreviations: CHD, coronary heart disease; DC, diabetic complications; DD, diabetes duration; HbA1c, hemoglobin A1c; FPG, fasting plasma glucose.

**Table S3. Correlation analysis of GSK-3β with clinical parameters.**

|  | | MMSE | Age | DD | HbA1c | FPG |
| --- | --- | --- | --- | --- | --- | --- |
| GSK-3β-S9 | R | 0.230 | -0.288 | -0.156 | 0.018 | 0.185 |
|  | *P* | <0.001 | <0.001 | <.001 | 0.582 | <0.001 |
| GSK-3β-Total | R | -0.117 | -0.229 | -0.108 | 0.113 | 0.262 |
|  | *P* | <0.001 | <0.001 | <.001 | 0.001 | <0.001 |
| GSK-3β-Total/S9 | R | **-0.494** | **0.123** | **0.094** | **0.146** | **0.068** |
|  | *P* | **<0.001** | **<0.001** | **0.002** | **<0.001** | **0.026** |
| N | | 1139 | 1139 | 1122 | 910 | 1079 |

*Notes*: Abbreviations: MMSE, Mini-Mental State Examination; DD, diabetes duration; HbA1c, hemoglobin A1c; FPG, fasting plasma glucose.

**Table S4. Linear regression analysis of association between other factors with GSK-3β activity**

| Variables | β | SE | 95% CI | *P* value |
| --- | --- | --- | --- | --- |
| ε2 | 0.040 | 0.094 | -0.143, 0.224 | 0.666 |
| **ε4** | **0.235** | 0.094 | 0.051, 0.419 | **0.012** |
| **Age** | **0.018** | 0.005 | 0.008, 0.027 | **0.000** |
| **Sex** | **-0.152** | 0.072 | -0.293, -0.010 | **0.036** |
| **Hypertension** | **-0.194** | 0.073 | -0.337, -0.051 | **0.008** |
| **Hyperlipidemia** | **0.178** | 0.089 | 0.004, 0.325 | **0.045** |
| CHD | 0.172 | 0.130 | -0.083, 0.427 | 0.186 |
| DC | 0.060 | 0.074 | -0.085, 0.205 | 0.415 |
| **DD** | **0.013** | 0.006 | 0.001, 0.024 | **0.029** |
| HbA1c | 0.024 | 0.021 | -0.017, 0.065 | 0.254 |
| **FPG** | **0.024** | 0.011 | 0.001, 0.046 | **0.039** |

*Notes*: ε3 was used as the reference. Analyses were performed with adjustment for age, sex, hypertension (Yes/No), hyperlipemia (Yes/No), CHD (Yes/No), diabetic complications (Yes/No), HbA1c, FPG, diabetes duration. ε3 was used as the reference. Abbreviations: CHD, coronary heart disease; DC, diabetic complications; DD, diabetes duration; HbA1c, hemoglobin A1c; FPG, fasting plasma glucose.
